# Monitoring of Freeze-Thaw Cycles in Concrete Using Embedded Sensors and Ultrasonic Imaging

**DOI:** 10.3390/s140202280

**Published:** 2014-01-29

**Authors:** Javier Ranz, Sofía Aparicio, Héctor Romero, María Jesús Casati, Miguel Molero, Margarita González

**Affiliations:** 1 Instituto de Tecnologías Físicas y de la Información “Leonardo Torres Quevedo”, ITEFI (CSIC), 28006, Madrid, Spain; E-Mails: sofia.aparicio@csic.es (S.A.); miguel.molero@csic.es (M.M.); m.g.hernandez@csic.es (M.G.); 2 Departamento de Ingeniería Civil: Construcción, E.T.S.I.C.C.P., U.P.M. 28040, Madrid, Spain; E-Mail: romerohectorl@hotmail.com; 3 Departamento de Vehículos Aeroespaciales, E.U.I.T. Aeronáutica, U.P.M. 28040, Madrid, Spain; E-Mail: mariajesus.casati@upm.es

**Keywords:** freeze-thaw cycles, monitoring, concrete, embedded sensors, ultrasound

## Abstract

This paper deals with the study of damage produced during freeze-thaw (F-T) cycles using two non-destructive measurement approaches—the first approach devoted to continuous monitoring using embedded sensors during the cycles, and the second one, performing ultrasonic imaging before and after the cycles. Both methodologies have been tested in two different types of concrete specimens, with and without air-entraining agents. Using the first measurement approach, the size and distribution of pores were estimated using a thermoporometrical model and continuous measurements of temperature and ultrasonic velocity along cycles. These estimates have been compared with the results obtained using mercury porosimetry testing. In the second approach, the damage due to F-T cycles has been evaluated by automated ultrasonic transmission and pulse-echo inspections made before and after the cycles. With these inspections the variations in the dimensions, velocity and attenuation caused by the accelerated F-T cycles were determined.

## 1. Introduction

Concrete structures located in cold climates suffer damage by freeze-thaw (F-T) cycles during their life cycle. The freezing and melting of water with deicing salt in porous structure causes serious damage and requires large investments in the repair and/or replacement of such structures. This deterioration process has widely been studied and as consequence different theories and standards have been proposed to evaluate the resistance of concrete subjected to F-T cycles using accelerated tests.

Among the theories proposed in the literature that describe the effect of water frost in the porous structure of cement-based materials the hydraulic and osmotic pressure theory proposed by Powers and Helmuth [1], and a micro ice lens model developed by Setzer [2], which takes into account the water and heat transport during micro-ice crystal formation, can be mentioned. The crystallization pressure of freezing water in pores was detailed by Scherer [3]. Penttala [4] derived material freezing deformation by effective freezing stress arising from crystallization pressure based on the thermodynamic equilibrium of phase change. Coussy and Fen-Chong [5] proposed a pore model, taking into account both viscous water flow and thermodynamic equilibrium between ice and capillary supercooled water. Their purpose was to describe the pore water cryosuction and stress relaxation during freezing. This model was later on developed into a comprehensive thermoporoelastic model for freezing cementitious materials [6,7]. The application of thermoporometric model requires measurements of temperature and the ice volume in pores during the material frost. Ice formation in pores can be determined by induced changes in physical properties or the enthalpy of fusion as low temperature calorimetry [8], nuclear magnetic resonance [9], differential scanning calorimetry [10], dielectric measurements [6,11], ultrasonic velocity measurements [12,13]. In this paper, ultrasonic measurements will be used to assess the ice volume in the pores.

Many standards were developed to evaluate the resistance of concrete subjected to accelerated F-T cycles, such as: UNE 12390-9 [14], ASTM C666/C666M-03 [15], prENV-9 [16], JIS A 1148-2001 [17], and Rilem TC 176-IDC [18,19], among others. However, these standards differ in the testing method used, the methodology used for evaluating damage and the evaluation criteria. In addition, none of these standards have been designed to perform the measurements as a real-time continuous monitoring process. The non-destructive testing methods more employed to assess damage in concrete are the ultrasonic velocity [15,20,21] and the fundamental transverse frequencies measurements [16,18,19].

This paper deals with the evaluation of the resistance of concrete to frost action by two non-destructive methodologies. In the first case, continuous measurements of temperature, humidity and ultrasound were performed during all the cycles while in the second one, automated inspections were made only before and after the cycles. This paper represents the first time, to the best of our knowledge, that the damage state has been evaluated monitoring several parameters at short time intervals (2 min) using embedded temperature/humidity sensors and ultrasonic transducers along the F-T cycles, and using ultrasonic imaging.

The first methodology is based on measurements of temperature and ultrasonic velocity during F-T cycles. Estimations of size and distribution of pores are carried out by using a thermoporometrical model [7]. The pore radius can be determined from the temperature measurements. The pore size distribution is estimated from the ice volume in the pores determined by relating ultrasonic velocity measurements and estimations of volume fraction using a micromechanical model developed by Hernández *et al.* [22]. The results obtained applying the thermoporometrical model has been compared with the measurements obtained using mercury porosimetry.

In the second methodology, automated ultrasonic inspections were made before and after the F-T cycles to obtain maps of dimensions, velocity and attenuation in all specimens.

The experimental trials were done using two types of concrete specimens with and without air entraining agents that were subjected to accelerated F-T cycles in accordance with the Rilem recommendations [19]. In this case the monitoring was performed using temperature, humidity and ultrasonic sensors. The temperature and relative humidity (T/RH) sensors were embedded in concrete during the manufacturing processes, while the ultrasonic sensors were adhered to the outer parallel surfaces of the specimens after the curing process. The embedded sensors, ultrasonic transducers and the multisensorial system for monitoring the specimens, were designed by the research group [23]. One advantage of the designed monitoring system is the large number of measurements obtained along F-T cycles without removing the specimens from the climatic chamber.

## 2. Study of Concrete Behaviour During the Freeze-Thaw Cycles

### 2.1. Thermoporometric Model

The thermoporometrical model allows the determination of pore diameter as well as the pore-size distribution in concrete. To make a thermoporometric measurement it is necessary to embed a liquid into the porous specimen, cool the specimen until all the liquid is frozen, and then warm it up until all liquid is melted again. This measurement can determine phase changes or the quantity of liquid/ice in the specimen. In the porous medium, water and ice in the pores can coexist, and the water can freeze when the interface liquid/ice is propagated towards the unfrozen zones. The model is based on the fact that the pore water freezes at a temperature determined by the curvature of meniscus at the interface solid/liquid according to the Gibbs-Thomson equation. It means that each pore diameter is related to a determined temperature as:

(1) $$P_{\text{cap}}=S_{f}(T-T_{0})+\left(T-T_{0}+\ln\frac{T_{0}}{T}\right)=\gamma \kappa$$

where P_cap_ is capillary pressure, *S_f_* is the entropy of fusion per unit of ice crystal volume (*S_f_* ≈ *1.2* MPa/K), *C_f_* is the heat capacity difference between water and ice per unit of ice crystal volume (*C_f_* ≈ *2.1* MPa/K) [24], T_0_ is the reference temperature (273.15 K for water) and *T* is the absolute temperature (*T* (K) = *θ* (°C) + *273.15*). In Equation (1) the parameters γ and κ denote the water/ice interface energy and the interface curvature, respectively. The water/ice interface energy can be determined as γ ≅ 36 + 0.25(T–T_0_) [25], while the interface curvature can be considered spherical or cylindrical and related to the radius of crystal ice, *r*, as:

(2) $$\begin{array}{c} \kappa =\frac{2}{r}(\text{spherical})\\ \kappa =\frac{1}{r}(\text{cylindrical}) \end{array}$$

Assuming that the crystal ice is surrounded by a homogeneous layer of unfrozen water, *r* is related to the pore radius *r_p_* and the layer thickness *e*, which remains constant and equal to 9Å as [24], by the relationship:

(3) $$r_{p}=r+e$$

At each temperature, the water contained in pores is frozen or melted, and can be related to a determined pore radius by:

(4) $$T_{f,m}^{*}={\left(P_{\text{cap}}\right)}^{-1}\left(\frac{2\gamma }{r_{p}^{f,m}-e}\right)$$

The logarithmic pore size distribution *ϕ*(*r_p_*) can be determined as the variation of ice volume fraction *S_c_* regarding the pore radius (temperature):

(5) $$\varphi (r_{p})=-\frac{\phi_{0}}{\rho }{\left(\frac{r_{p}}{r_{p}-e}\right)}^{3}\frac{\partial S_{c}}{\partial \log(r_{p})}$$

The above Equations (1)–(5) are used to obtain the pore size distribution from the ice volume formed in porous structure during F-T cycles, but it is necessary to know the limitations of this method before comparing it with other measures of porosity.

**Figure 1.**
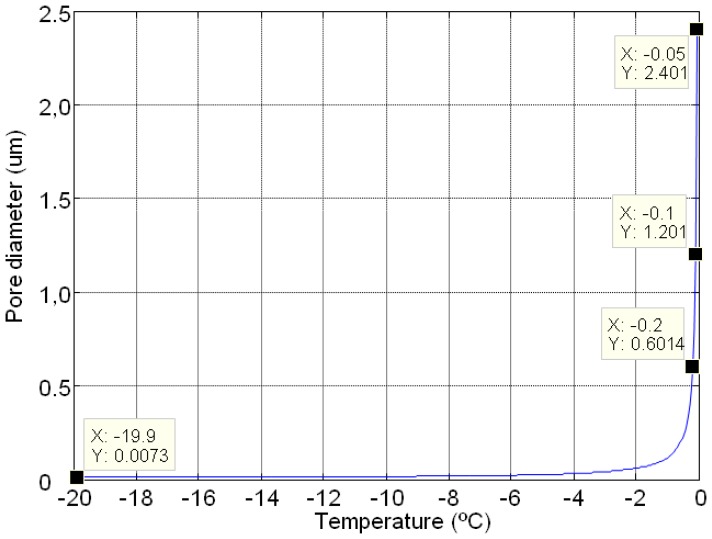
Numerical study of the theoretical behaviour of the pore diameter *versus* temperature.

**Figure 2.**
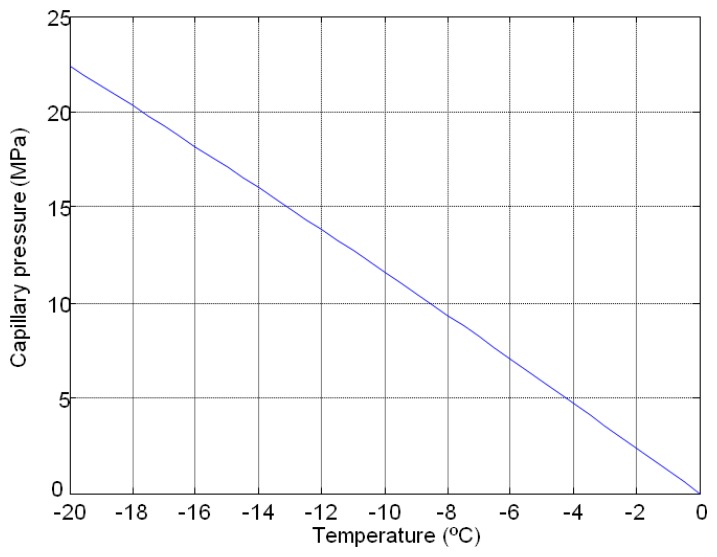
Numerical study of the theoretical behaviour of the capillary pressure *versus* temperature.

The variations in the produced capillary pressure and the pore diameters during a freezing and thawing cycle are calculated from Equations (1) and (2). In this case an F-T cycle between −20 °C to 0 °C with a step of 0.05° of temperature was used to establish the limitation of thermoporometrical model. Figures 1 and 2 show the behaviour of pore diameter and capillary pressure *versus* temperature, respectively. In Figure 1, the numerical behaviour of the pore diameter with the F-T cycles showed a vertical asymptote at 0 °C that implies limitations on the measurement.

The maximum possible capillary pressure to achieve in the freeze-thawing cycle is 22 MPa, and the range of pore diameters is between 7 nm and 2.4 μm. These limitations can be avoided if we increase the temperature of freezing and the precision of climatic chamber, e.g., considering a temperature of −100 °C and a precision of 0.001 °C, the range of pore diameter varies between 2 nm and 120 μm.

### 2.2. Determination of Ice Volume

Frozen water modifies the porous structure in several aspects such as geometry, density and elastic properties affecting global properties of the material. This behaviour is studied by a micromechanical model based on the Mori-Tanaka homogenization scheme [22,26,27]. This model takes into account microstructural characteristics of the constituent phases. From the formulation described in [22], the elastic constant tensor *C*, is obtained as:

(6) $$C=C^{m}+\nu^{p}\left(C^{pm}\right)\langle T_{p}\rangle {\left[v^{m}I+v^{p}\langle T_{p}\rangle +v^{a}\langle T_{a}\rangle \right]}^{-1}+v^{a}\langle C^{am}\rangle \langle T_{a}\rangle {\left[v^{m}I+v^{p}\langle T_{p}\rangle +v^{a}\langle T_{a}\rangle \right]}^{-1}$$

where *v* is the volume fraction, T represents Wu's tensor in global coordinates for each type of inclusion, and the brackets 〈〉 denote the average over all possible orientations. The superscripts *m*, *p* and *a* refer to the constituent phases, namely, matrix, and two types of inclusions, in this case pores and aggregates, respectively.

The tensor T is a function of the geometry, the distribution and the orientation of the inclusions and it can be calculated using the method given in [26]. The geometry of the inclusions is described by the Eshelby tensor [28] and is modelled in the micromechanical model by the aspect ratio *α* which is described by an ellipsoid.

The volume fraction of matrix can be calculated as *v^m^* = 1–*v^p^*–*v^a^*: When the temperature drops below 0 °C the water in the porous structure does not freeze at the same time, part of the water freezes and the rest can be super-cooled depending on the pores size [2–5]. For this reason the change in the volume fraction of water in ice in the porous structure is studied considering that this change follows a linear relationship:

(7) $$\begin{array}{cc} v^{w}=v^{p}-v^{i}; & v^{i}= \end{array}(0\vec{\leftarrow }v^{p})$$

where *v^w^* and *v^i^* denote the volume fraction of water and ice, respectively. It is worth noted that these variables have an opposite behaviour, when *v^i^* increases; *v^w^* decreases.

Considering the concrete as an isotropic material, the elastic tensor *C* is reduced to two independent elastic constants, *C*_11_ and *C*_44_ in reduced notation. These constants are related to the longitudinal (*V_l_*) and transverse (*V_t_*) velocities as:

(8) $$\begin{array}{cc} V_{l}=\sqrt{\frac{C_{11}}{\rho }} & V_{t}=\sqrt{\frac{C_{44}}{\rho }} \end{array}$$

The concrete density, *ρ*, is calculated in terms of density and volume fraction of constituent phases as:

(9) $$\rho =\rho^{p}v^{p}+\rho^{m}v^{m}+\rho^{a}v^{a}$$

A theoretical study was made to study the behaviour of the ultrasonic velocity with the increase of ice volume fraction in the pores. Before applying the micromechanical model it is necessary to take into account several assumptions. The concrete is considered as a three phase material composed by a solid matrix (cement paste without pores, *m*), aggregates (*a*) and pores (*p*) which are distributed randomly in the matrix. The sand and gravel are modelled as a one phase (aggregates) because they have the same elastic properties and previous studies have shown that the shape of the aggregates has little influence on the ultrasonic velocity [27]. Accordingly the geometry of aggregates is modelled as spheres (*α_a_* = 1). The geometry of pores is modelled as cylinder (*α_p_* = 1,000) representing capillary pores.

The elastic properties of non-porous cement paste matrix are estimated through an optimisation process described in [29] while the volume fractions of different phases are estimated from the mix proportions and the porosity of the specimens under study. The volume fraction of aggregates is equal to 0.69 for both groups of concrete specimens while the pores volume fraction is equal to 0.15 and 0.18, for groups A and B respectively. The properties of constituent phases of concrete studied in this work are outlined in Table 1.

**Figure 3.**
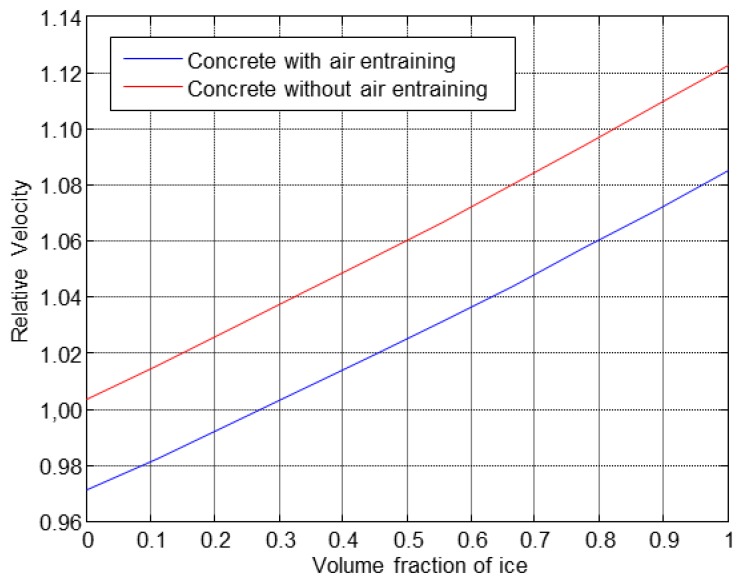
Theoretical study of the behaviour of the ultrasonic velocity *versus* ice volume.

The results obtained from the theoretical study about the behaviour of the ultrasonic velocity with the increase of the ice fraction volume are shown in Figure 3. An ice volume fraction equal to 0 corresponds to the saturated water specimen at the beginning of the first cycle, while a volume fraction equal to 1 corresponds to the specimen with all pores frozen.

The obtained results showed that the freezing of all volume of pores would provoke an increase of velocity around 10%, approximately, in both materials, see Figure 3. This fact will be verified with the experimental results.

## 3. Experimental Procedure

### 3.1. Materials

Two groups of concrete specimens, HA-30, were prepared with and without air-entraining agents, A (0.10% in mass) and B, respectively. All specimens were made with Portland cement CEM I 42.5 R. The aggregates used were silica sand and siliceous gravel river with sieve modules of 2.87 and 7.31, respectively. The maximum size of coarse aggregate is 25.4 mm. The proportions of the mixtures are shown in Table 2. A polycarboxylic type superplasticizer, SIKA Viscocrete 20 HE, was used to reduce the content of water. An air entraining agent, Sika AER 5, was used in concrete specimens (Group A) to improve the resistance of concrete to F-T cycles.

The specimens were manufactured in accordance with the standard UNE-EN 12390-2 [30]. In each mixture five cubic molds of 150 mm ×150 mm ×150 mm with a polytetrafluoroethylene thin plate were used, following the standard UNE CEN/TS 12390-9 EX [14]. Therefore, ten specimens of 150 mm ×150 mm ×70 mm of each group were produced. The air content of concrete was determined when the mixing procedure was completed. The curing process consists of storing the specimens in water during 7 days after demolding following the standard previously mentioned. After that, the specimens were introduced in a climatic chamber at 20 °C during 21 days to ensure the surface drying. After the curing process the lateral faces of the specimens were sealed in order to hinder moisture exchange with the environment.

### 3.2. Microstructural Testing

Microstructural testing of mercury intrusion porosimetry and thermogravimetric analyses were performed before and after the F-T cycles to obtain the porosity, pore size distribution and degree of hydration [31]. The open porosity of specimens was obtained from ASTM D4404-84 using a Microporomeritics Autopore IV 9500 instrument (from 0.07 to 230 MPa) allowing the determination of pore size between 6 nm and 175 μm. The characterization of hydrated products was made in a Setaram, Labsys Evo model, thermal analyser, with a precision balance of 0.1 μg.

### 3.3. Freeze-Thaw Cycles

**Figure 4.**
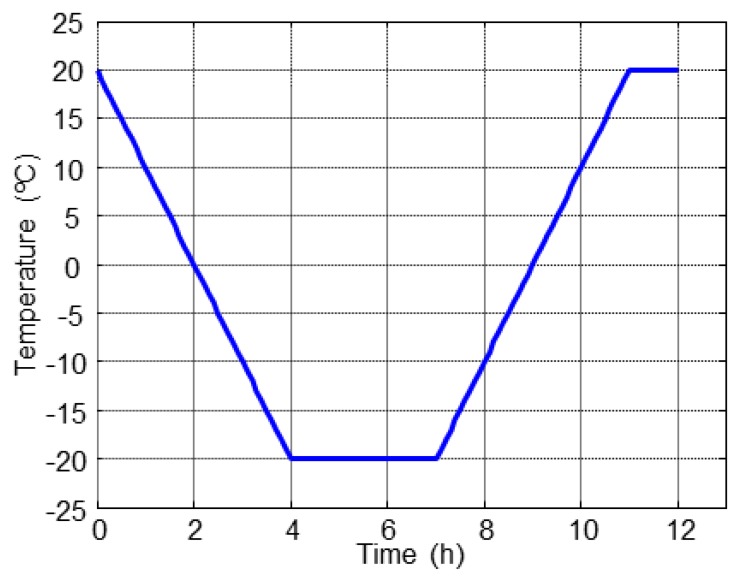
Accelerate freeze-thaw cycle.

The accelerated F-T cycles (F-T) were made according to the standard UNE-CEN/TS 12390-9, CDF Test (Alternative Method) [14], in a DYCOMETAL CCK-40/1000 climatic chamber. The specimens were partially immersed in a 3% NaCl dissolution at depth of 3 mm and subjected to repeated F-T cycles, in total 28 cycles. The applied F-T cycle is shown in Figure 4.

### 3.4. Monitoring of Freeze-Thaw Cycles

**Figure 5.**
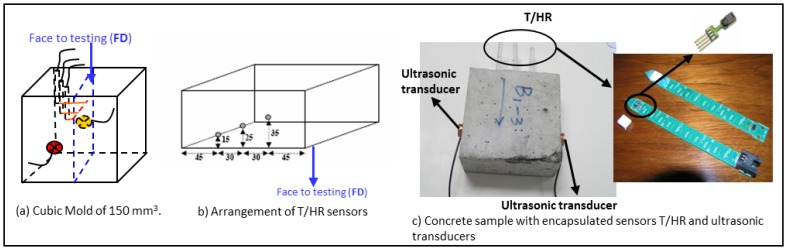
Arrangement of T/RH and US sensors in concrete specimens.

In this paper the concrete resistance to frost was evaluated by monitoring the temperature, relative humidity and ultrasonic parameters along all F-T cycles. Temperature and relative humidity sensors were embedded in the concrete while the ultrasonic transducers were adhered to the outer side surface before the lateral faces were sealed with epoxy paint according to the standard [14], see Figure 5. Therefore, a large number of measurements along the F-T cycles were obtained without removing the specimens from the climatic chamber.

Commercial sensors, SHT1 from Sensirion Compay, were used to measure temperature and relative humidity. They were placed at different depths (15, 25 and 35 mm) of the concrete specimens through the specimen face in contact with the dissolution of NaCl, Figure 5b. A sensor with a commercial filter cap, SM1 of Sensirion Company, was incorporated into a special device designed by the research group [23]. These devices are based on a printed circuit board with an integrated sensor. They have the advantage of knowing the depth to which this sensor was embedded, see Figure 5c. The T/RH sensors were embedded in concrete specimens during the filling of the mold, before the vibration of the last layer of material. Additionally, three TH03 sensors by Pico Technology were used to monitor the temperature and relative humidity inside the climatic chamber.

The ultrasonic transducers (US) were designed and manufactured by the research group due to the size of the concrete specimens and the conditions of the trial. Piezoelectric ceramic with a central frequency of 500 kHz, emitting in longitudinal mode were used to manufacture the ultrasonic transducers. These ceramics were encapsulated in a ceramic body to be electrically insulated and to improve the signal noise ratio. The measurements were made in transmission mode with the transducers adhered to the outer side surface approximately at 35 mm of the face in contact with the dissolution, see Figure 5c.

**Figure 6.**
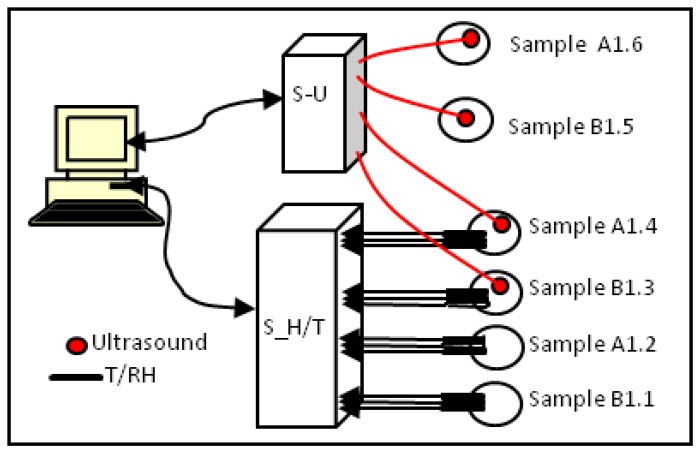
Multi-sensorial system for monitoring.

A multi-sensory system of real-time monitoring in Matlab environment was designed allowing the simultaneous acquisition of up to four channels for ultrasonic measurements and 20 channels for T/RH measurements with programmable acquisition intervals. Figure 6 shows the sensing scheme used and the specimens monitored in this paper. “S_H/T” is the commercial system EK-H3 of Sensirion Company that collects data from 12 embedded sensors. “S-U” is the ultrasonic NURVE 4 four-channel system, which monitors simultaneously two specimens of each mixture, at sampling frequency of 10 MHz. The NURVE system was used to generate and receive the A-scan signals. Using these signals the time-of-flight was measured to determine the phase velocity.

The ultrasonic velocity (*V*) was determined by the travelling time of the ultrasonic pulses in the specimen (*t*), the thickness (*d* = 150 mm) considered constant along all cycles and the reference travelling time (*t_0_*) in order to calibrate the ultrasonic acquisition system using a reference aluminium specimen:

(10) $$V=\frac{d}{t-t_{0}}$$

The travel time, t, from the A-scan signals was calculated using a zero-crossing algorithm. This algorithm determines the first zero-crossing point of the time-domain signal according to a given threshold, e.g., 10% in reference to the normalized amplitude. Further details can be found in [32].

In summary, three specimens of each mix were monitored, see Figure 6, and only in two of these specimens, A1.4 and B1.3, were all sensors (T/RH and US) located. The measurements of temperature, relative humidity and ultrasonic parameters were taken every 5 min along 28 F-T cycles, 12 measurements per hour, 144 per cycle and in total 4,032 measurements for each specimen.

### 3.5. Ultrasonic Automated Inspections

Automated ultrasonic inspections providing maps of dimensions, velocity and attenuation of all specimens used in the trial were made before and after the F-T cycles to evaluate their quality. The inspection before the cycles has a two-fold purpose: on the one hand the heterogeneity effect of concrete from the possible deterioration caused by the F-T cycles should be decoupled, and on the other hand, it should be checked that the embedded T/RH sensors do not affect the propagation of ultrasonic waves through the concrete specimens. The maps obtained after the cycles give the damage state of the specimens which are compared with the monitoring results.

**Figure 7.**
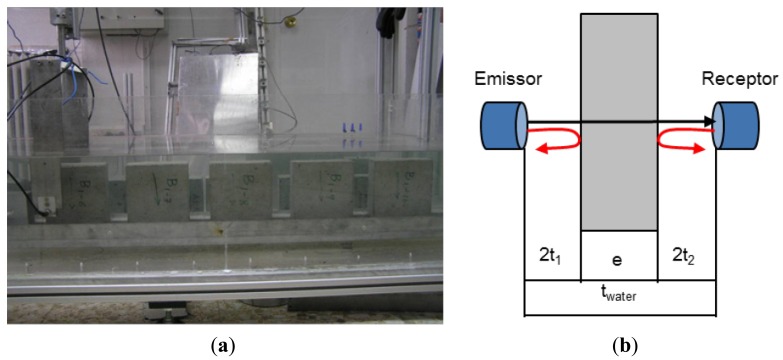
(**a**) Automated inspections of concrete specimens; (**b**) Schematic representation of ultrasonic inspections, transmission (black) and pulse-echo (red).

The automated inspections in immersion perform a uniform insonification during the scanning by the ultrasonic transducers, involving precise measurements as well as optimising the number, distribution, and duration of the inspection. Two types of inspections were conducted simultaneously, in through-transmission and pulse-echo mode, to determine dimensions, velocity and attenuation measures. The specimens were scanned on two faces: width and length, with a standard automatic system of three Cartesian axes, see Figure 7a. The specimens were aligned at the bottom of the tank and two ultrasonic transducers scanned the parallel surfaces of the specimens with a spatial resolution of 5 mm in horizontal and vertical directions, with the same frequency as those used in the continuous monitoring (500 kHz) and a frequency sampling of 20 MHz. Approximately 900 and 450 A-scans were obtained in length and width, respectively, on each specimen. The thickness of the specimen is determined as:

(11) $$e=V_{\text{water}}\cdot (t_{\text{water}}-t_{1}-t_{2})$$

where *e* is the thickness of concrete, *t_water_* is the travelling time in water without the specimen, and *V_water_* is the velocity in water at inspection temperature, see Figure 7b.

The ultrasonic velocity of the concrete specimens was determined as:

(12) $$V=\frac{e}{t-t_{\text{water}}+\frac{e}{V_{\text{water}}}}$$

where *t* is the travelling time of the signal through the specimen. The attenuation coefficient (*α*), expressed in dB/m, was computed as the quotient of the maximum amplitude of the received pulse traveling through the specimen, *A_s_*, and the pulse travelling just in water, *A_w_*, as:

(13) $$\alpha =20\log_{10}\left(\frac{A_{s}}{A_{w}}\right)$$

From the automated inspections, dimensions, velocity and attenuation maps were generated for the concrete specimens, before and after the cycles. It is necessary to point out that all manufactured specimens (10 specimens of each mix) were inspected, although not all were subjected to accelerated cycles in the climatic chamber.

## 4. Experimental Results

### 4.1. Microstructural Testing Results

**Figure 8.**
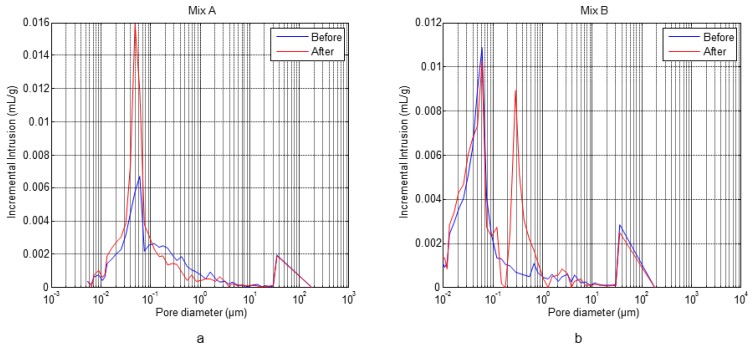
Pore size distribution of concrete before and after the cycles: (**a**) without air entrainment, (**b**) with air entrainment.

The obtained results from the microstructural mercury intrusion porosimetry and thermogravimetric analysis tests performed before and after the F-T cycles for specimens A1.4 (Mix A) and B1.3 (Mix B) are shown in Figure 8 and Table 3, respectively, [31].

Figure 8 shows the pore size distribution of concrete with and without air entrainment, A1.4 and B1.3, respectively. The specimen A1.4 shows a maximum in the pore distribution centred in 0.05 μm, before the cycles. However, the pores volume is increased as consequence of freeze and thaw of water after the cycles (see Table 3). Also, a new peak appears at 40 μm after the F-T cycles. In the specimen B1.3 after the F-T cycles a new peak at 0.2 μm appears in the zone of capillary pores, with a volume similar to the peak at 0.05 μm, which is present before and after the cycles. This behaviour indicates the formation of microcracks in concrete without air entrainment.

In both concrete types the porosity is increased after the F-T cycles, see Table 3. According to the thermogravimetric analysis results, see Table 3, it is observed that in both types of concrete the degree of hydration increases after the cycles. This behaviour might be a consequence of the existing low humidity during the curing process conditions and the contribution of water during the F-T cycles, similar behaviours were also observed by others authors [33,34].

### 4.2. Temperature and Relative Humidity Monitoring

**Figure 9.**
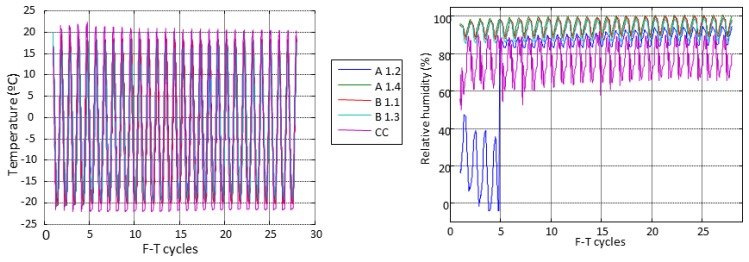
Temperature and relative humidity along cycles.

The temperature and relative humidity behaviour inside the concrete specimens and the ambient conditions in the climatic chamber along all F-T cycles is represented in Figure 9. The dissolution of NaCl strongly affects the humidity sensors of the specimen A1.2 as shown in Figure 9 (right). The temperature and relative humidity inside of specimens follow the same behaviour along of the F-T cycles, according to the fixed cycle in Figure 4.

**Figure 10.**
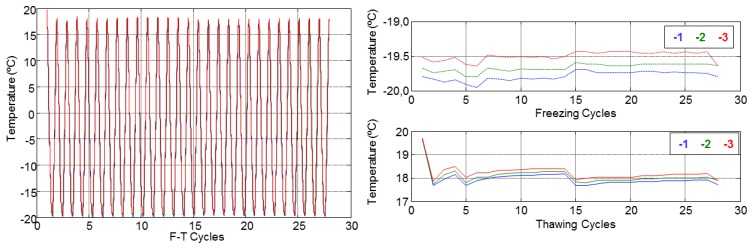
Temperature inside specimen A1.4.

**Figure 11.**
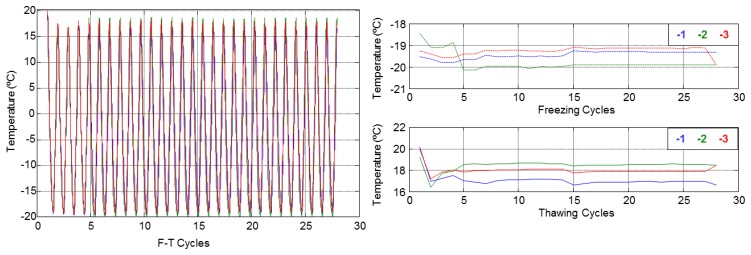
Temperature inside specimen B1.1.

The temperature behaviour with respect to depth inside the specimens and the maximum and minimal values during all F-T cycles are shown in Figures 10 and 11. These figures show that temperature does not vary with depth. Therefore, the mean of three sensors was used for each specimen.

**Figure 12.**
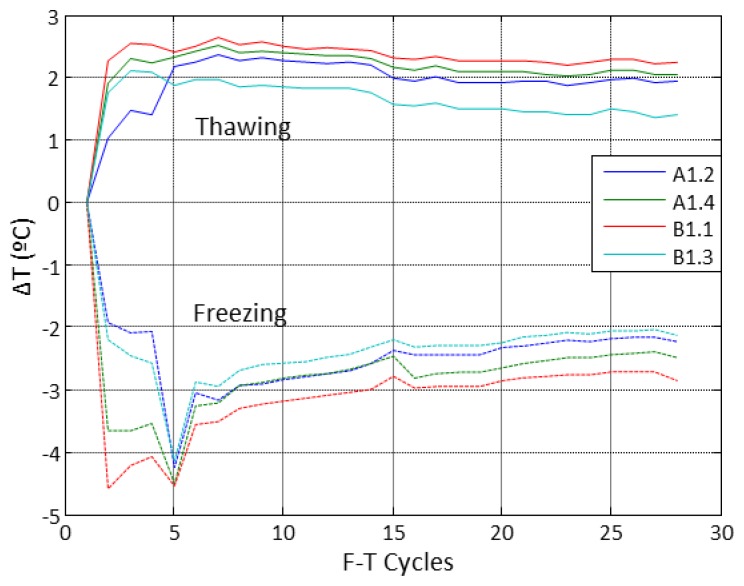
Difference of temperature between concrete specimens and climatic chamber.

Figure 12 shows the differences of temperature between the sensors embedded in the concrete specimens and the temperature inside the climatic chamber. These differences were calculated at the plateau of the F-T cycles. The highest differences appeared during the first five F-T cycles, probably due to the thermal inertia of the concrete. After the first five cycles the temperature behaviour is almost constant and in general significant differences are not observed among them. We can conclude that the temperature inside the concrete specimens follows the F-T cycle settings in the climatic chamber.

**Figure 13.**
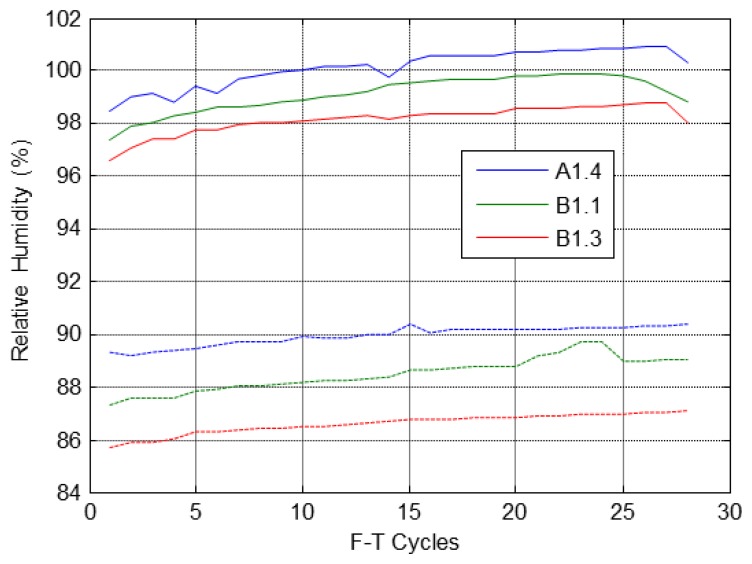
Relative humidity at the freezing (-) and thawing (--) plateaus.

Figure 13 shows the behaviour of the relative humidity during the cycles and with respect to depth inside specimens during the F-T cycles. It can be appreciated that in all specimens the humidity increases with the F-T cycles.

### 4.3. Monitoring of Ultrasonic Parameters

**Figure 14.**
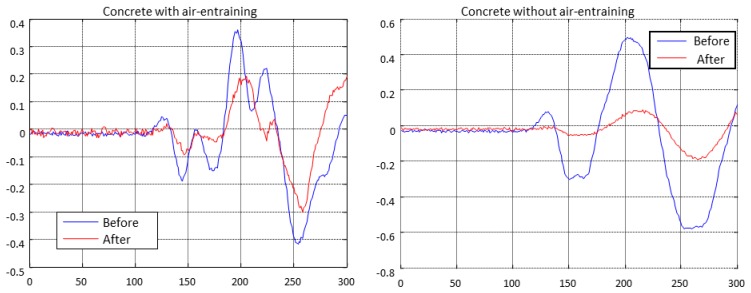
A-scan of concrete specimens, with and without air entrainment, before and after the cycles.

The ultrasonic signal, A-scan, of a specimen of concrete with and without air entrainment, before and after of the F-T cycles is shown in Figure 14. In both cases, it is observed that the ultrasonic pulse changes and the amplitude decrease strongly after the cycles. This behaviour might lead to errors in the ultrasonic velocity values obtained using conventional equipment if a threshold algorithm was used to measure the travelling time [21].

**Figure 15.**
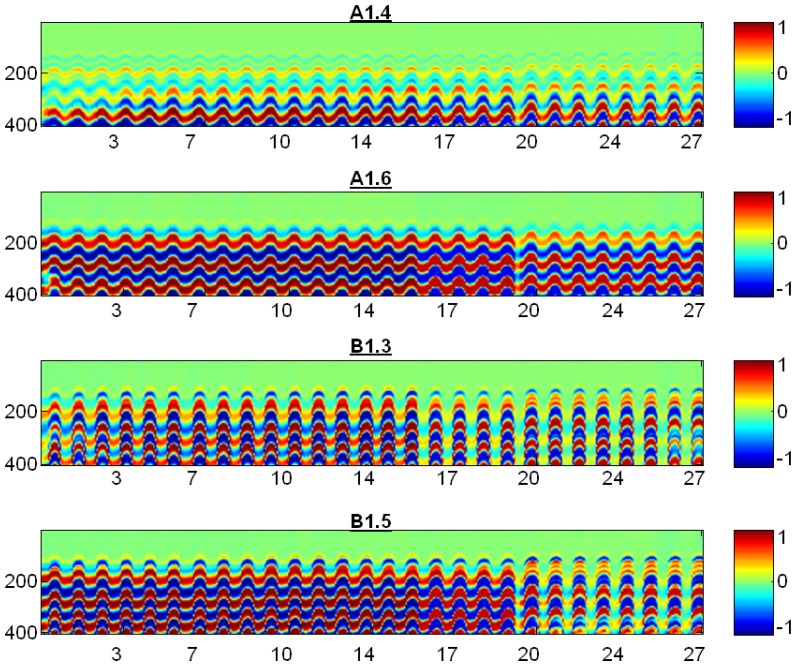
B-scan images of concrete specimens along of the cycles.

A B-scan image was generated from all recorded A-scan signals throughout the cycles (see Figure 15), this is a false colour image where the colour represent the amplitude of ultrasonic signal. The number of F-T cycles and the sampled signal for the A-scan are shown in the x- and y-axis respectively.

In all specimens it can be observed that in the 20th F-T cycle there is a slight discontinuity in the measurements, shown as a mild colour change, due to an electric power failure in the laboratory. In specimen B1.3 a change can be seen in the ultrasonic signal from the 16th and 17th F-T cycles indicating damage in the concrete material. A similar but smoother behaviour was also observed in specimen B1.5.

**Figure 16.**
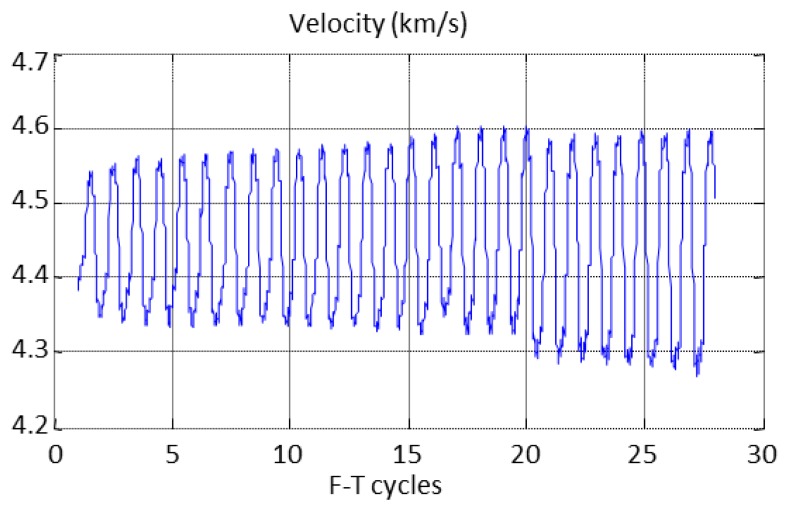
Monitorization of the ultrasonic velocity during the F-T cycles.

The behaviour of the ultrasonic velocity along the cycles for specimen A1.4 is shown in Figure 16. It can be seen that the velocity measurements follow the F-T cycles, increasing as the temperature decreases and *vice versa*. The same behaviour was observed in all monitored specimens and the relative velocity was determined to analyse this behaviour.

**Figure 17.**
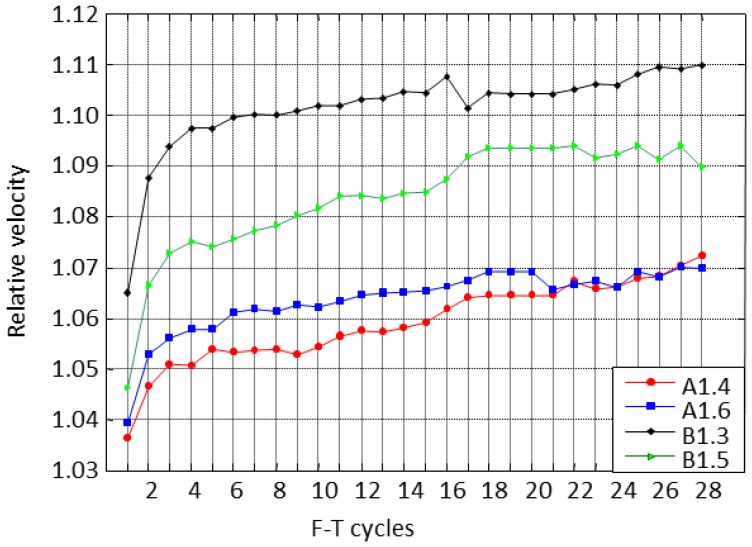
Relative velocity of specimens at the plateau of the cycles.

The relative velocity was calculated as the ratio of the maximum and minimum velocity at the plateau of freezing and thawing cycles, respectively. The relative velocity along the F-T cycles illustrates that the specimens are grouped by the type of mixtures, A or B, see Figure 17.

In all specimens the relative velocity increases progressively along the cycles, probably indicating damage in the material. The concrete specimens with air entrainment exhibit the smallest differences among frozen and unfrozen states. However, the difference between the F-T cycles increases more with the cycles for the concrete specimens without air entrainment. The B1.3 specimen has the higher relative velocity indicating that it is the most damaged specimen. This behaviour is consistent with the previous results obtained in Figure 15. The increased relative velocity is a consequence of the increase in the ice pore volume and consequently a raise in porosity. An increase in velocity regarding the first cycle is observed. This behaviour will be studied subsequently with the thermoporometric model.

**Figure 18.**
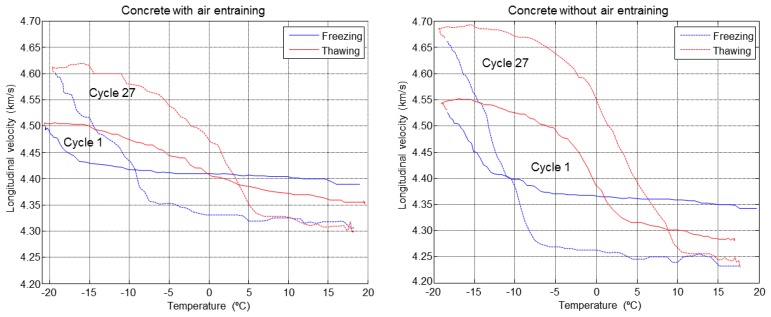
Behaviour of ultrasonic velocity in F-T cycles, 1 and 27.

The evolution of the ultrasonic velocity with the temperature in the concrete specimens is shown in Figure 18. In this case only F-T cycles 1 and 27 are represented. It can be noticed that the velocity in mixture B is higher than the mixture A due to the air entraining agents which cause an increased attenuation producing a decrease in velocity. It is also observed how the hysteresis increases between the freeze and melting between the first and last F-T cycles, this effect is more pronounced in the concrete without air entrainment indicating that there is more ice volume in these specimens.

### 4.4. Determination of Volume Fraction of Ice

**Figure 19.**
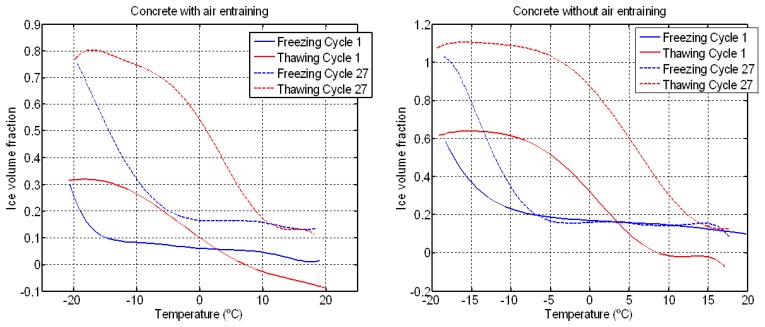
Variation of volume of ice during the F-T cycles from the micromechanical model and ultrasonic velocity.

The volume fraction of ice was determined from the micromechanical model and the longitudinal velocity. The variation of ice volume fraction with temperature is shown in Figure 19 at the beginning and end of cycles. In both materials, an increase in the ice volume and the hysteresis between freezing and thawing is observed, impying that the volume of frozen pores increases. The values of ice volume fraction greater than 1 in concrete without air entrainment (cycle 27) are due to the porosity increase as can be seen in Table 3. In concrete with air entrainment, the ice volume fraction is less than 0 in cycle 1 because the ultrasonic velocity during thawing is less than the minimum velocity during freezing. This behaviour may indicate that the concrete sample has been microcracked and, hence, the velocity decreases.

### 4.5. Application of Thermoporometry to Determine the Pore Size Distribution in Concrete

**Figure 20.**
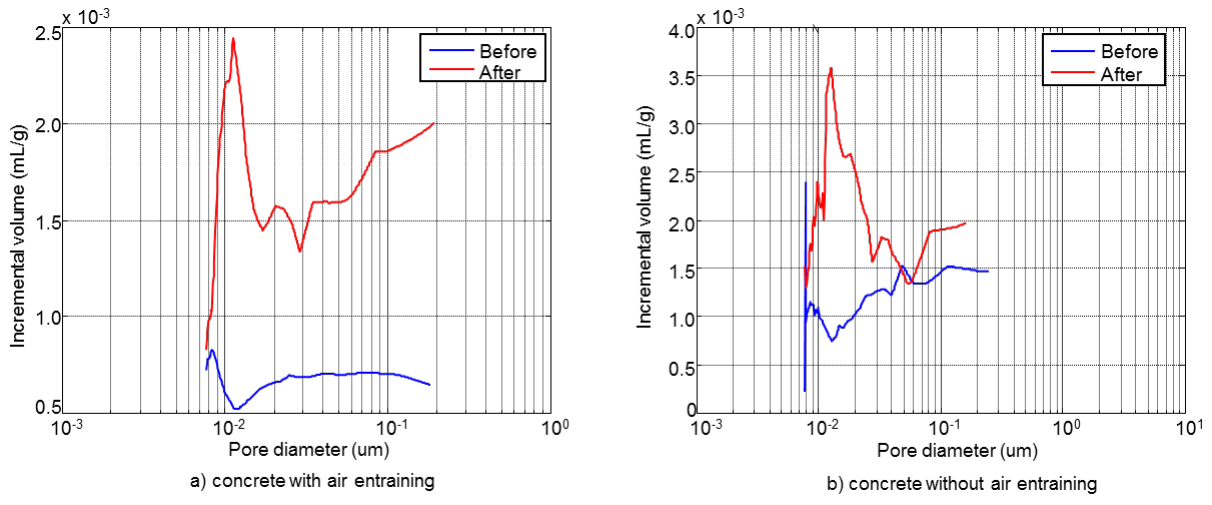
Pore size distribution of concrete obtained by thermoporometry.

**Figure 21.**
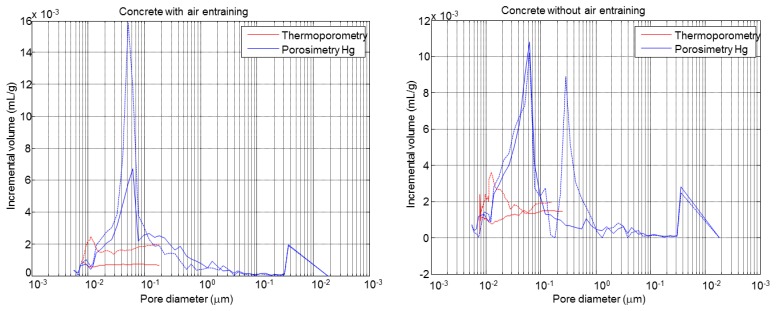
Distribution of pore size from mercury porosimetry and thermoporometrical model (solid line means “before cycles”; dashed line means “after cycles”).

The pore size distribution of concrete specimens determined by the thermoporometry technique is shown in Figure 20. In both materials, the F-T cycles led to an increase in the volume and diameter of pores and to a peak at 0.01 μm. This behaviour is more accentuated in concrete without air entrainment. Figure 21 shows the comparison between the pore size distribution determined by mercury porosimetry and thermoporometry techniques before and after the F-T cycles. In both cases an increase of the pore volume is observed, but in different proportions. This is a consequence of the differences between these methodologies (mercury/water, high pressure/capillarity and thermal expansion) and the test conditions. In the first case, the samples are dried under vacuum while with the thermoporometry the samples are saturated.

Also, the difference between the exerted pressure by mercury porosimetry and thermoporometry techniques is approximately of 7.5 in our experiments. Similar behaviours have been pointed out by other authors [34,35]. Despite the differences between these methods it it should be noted that the use of ultrasonic and thermoporometry techniques can be an alternative method for characterising F-T cycles in cementitious materials.

### 4.6. Ultrasonic Images of Concrete before and after the F-T Cycles

**Figure 22.**
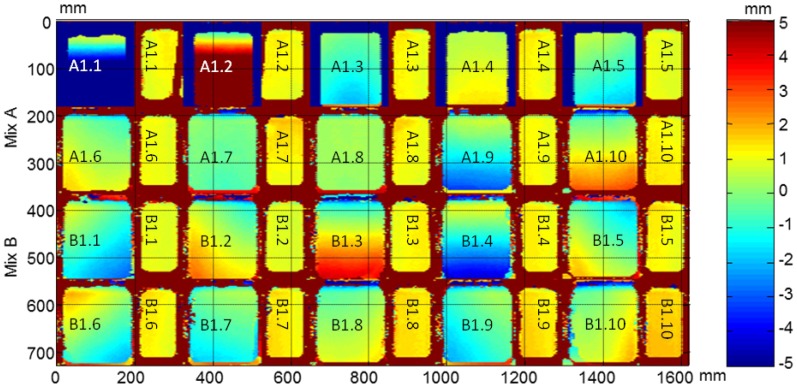
Dimensions of concrete specimens obtained from the ultrasonic inspections before the F-T cycles.

**Figure 23.**
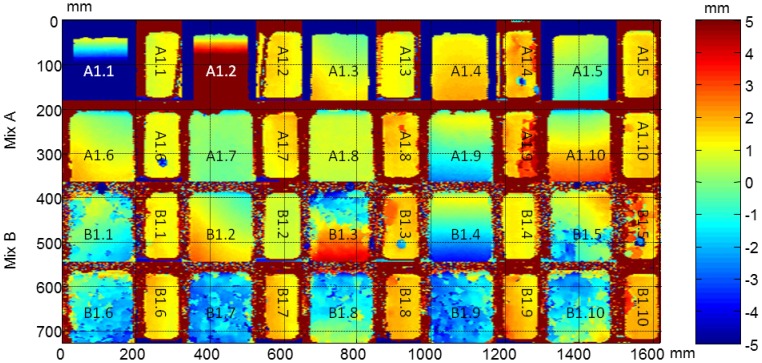
Dimensions of concrete specimens obtained from the ultrasonic inspections after the F-T cycles.

**Figure 24.**
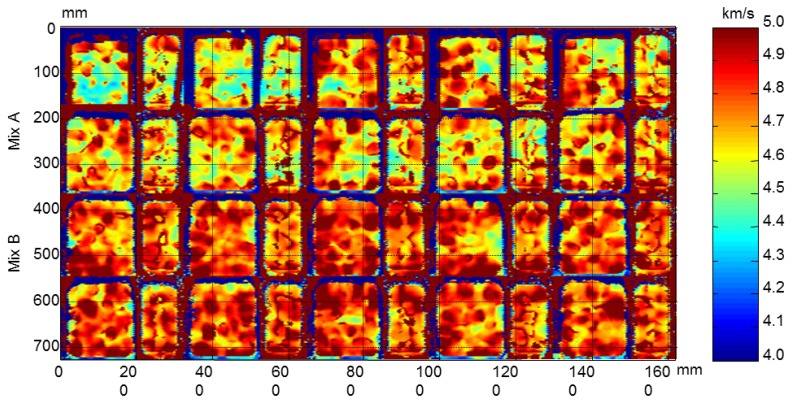
Velocity maps of concrete specimens before the cycles.

**Figure 25.**
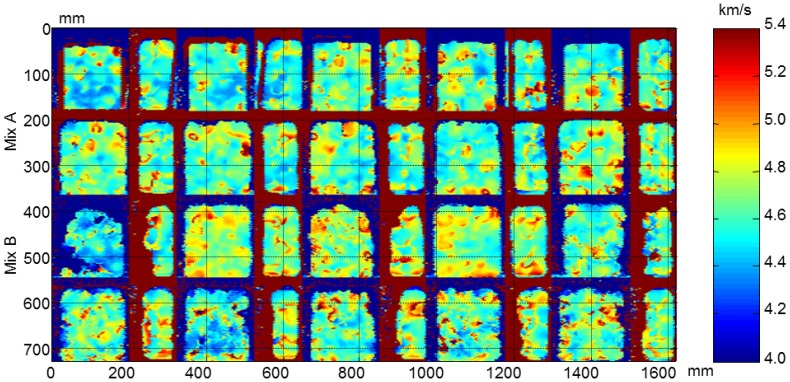
Velocity maps of concrete specimens after the cycles.

**Figure 26.**
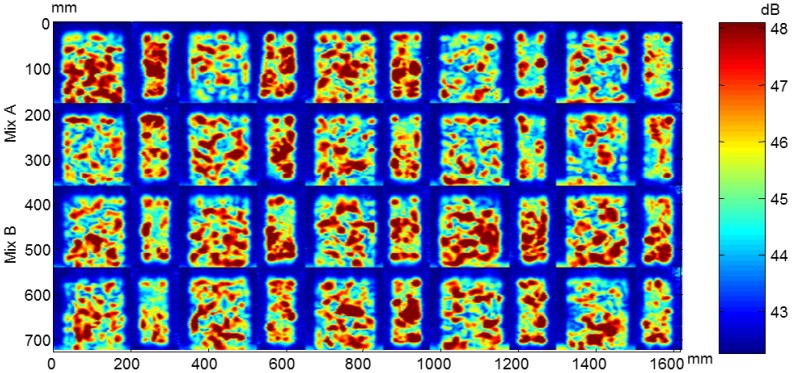
Attenuation maps of concrete specimens before the cycles.

**Figure 27.**
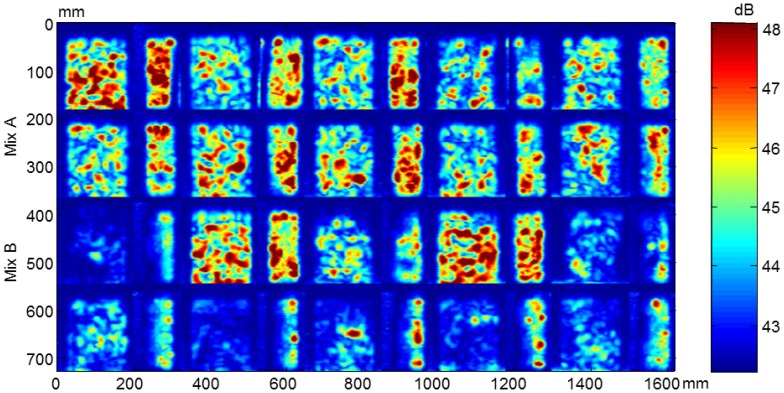
Attenuation maps of concrete specimens after the cycles.

From the ultrasonic automated inspections three parameters were determined: dimensions (width and length), ultrasonic velocity and attenuation for each specimen before and after the F-T cycles. The maps of dimensions, velocity and attenuation before and after the cycles are show in Figures 22, 23, 24, 25, 26 and 27.

It should be noticed that in all images the second and fourth specimens in the third row, B1.2 and B1.4, are not subjected to the accelerated cycles. The dimension maps before and after the F-T cycles of all specimens and the arrangement of specimens in automated inspections are shown in Figures 22 and 23, respectively. These images indicate the difference of dimensions regarding nominal, width = 70 mm and length = 150 mm in both Figures. The differences with the nominal dimensions before the cycles reaches up to 5 mm. It is probably due to movement of the polytetrafluoroethylene thin plate in the filling process of the cubic mold.

In the images after the F-T cycles, see Figure 23, a non-uniform degradation is observed in both types of concrete, A and B. In this Figure, some circles of blue colour appear in the specimens A1.4, A1.6, B1.3 and B1.5 (at the 70 mm ×150 mm face), which are the adhered ultrasonic transducers placed on the monitored specimens. It should be noted that these circles are not observed in the images before the cycles because they were glued on the specimens afterwards. It can be seen that the concrete specimens without air entraining agents are more degraded than the specimens with air entraining agents and the edges of the specimens are not defined. It can be seen that the B1.3 specimen is the most damaged. These results are in agreement with the surface mass loss results, 3.23 kg/m^2^ for specimens of Mix B and 0.10 kg/m^2^ for specimens of Mix A [31].

The velocity maps of the concrete specimens before and after the F-T cycles are shown in Figures 24 and 25, respectively. In the images before the cycles, the non-uniformity of the ultrasonic velocity within the same type of concrete specimens can be observed. The concrete specimens with air entraining agents have also less velocity that the specimens without air entrainment due to the air bubbles causing a decrease in ultrasonic velocity [36].

After the F-T cycles a decrease in velocity is observed in all specimens, Figure 25. This behaviour is more accentuated in concrete without air entrainment which edges are not well defined. In the edges of these specimens the damage is higher and diffraction effects in ultrasonic wave propagation occur due to the loss of material, resulting in erroneous travelling time measurements. It can be seen that the B1.3 y B1.5 specimens are more degraded that the A1.4 and A1.6 specimens. This behaviour coincides to the velocity monitoring results.

The attenuation maps of concrete specimens before and after the cycles are shown in Figures 26 and 27, respectively. The non-uniformity of concrete before the cycles is observed in both types of concrete. The decrease of amplitude in concrete without air entrainment after the cycles is due to the damage. The attenuation behaviour agrees with the velocity results.

## 5. Conclusions

In this paper the damage produced in concrete during F-T cycles was evaluated using two non-destructive approaches, continuous measurements of T/RH and ultrasonic velocity during all the cycles, and automated inspections only before and after the cycles.

The continuous monitoring of two types of concrete, with and without air entraining agents, during the freeze-thaw cycles was carried out using temperature, relative humidity and ultrasonic sensors. These embedded sensors and the multisensorial system used for the monitoring were developed by our research group. The monitoring of T/RH was performed at different depths. The behaviour of temperature and humidity were in phase during the cycles and it was observed that it did not vary with depth. It was also verified that the temperature inside the concrete specimens follows the F-T cycle fixed in the climatic chamber. Concrete uniformly freezes from the first cycle.

From the ultrasonic wave monitoring, it was observed that the ultrasonic pulse changes and the amplitude decreases strongly after the cycles in both materials. In addition, the relative velocity was calculated at the plateau of F-T cycles, and in all monitored specimens it was observed that this velocity increases progressively along the cycles, allowing the identification of the beginning of damage from the first cycle. The increment of relative velocity is a consequence of the increase in the ice pore volume and the porosity. An increase in velocity regarding the first cycle is observed.

A thermoporometrical model was used to estimate the size and distribution of pores using temperature and ultrasonic measurements. The results obtained show that the volume and diameter of pores increase after the cycles in both materials, although is more accentuated in concrete without air entrainment. This behaviour agrees with the results obtained with mercury porosimetry technique, but in different proportions as a consequence of the differences between these methodologies.

The automated ultrasonic inspections were made before and after the F-T cycles to decouple the effect of concrete heterogeneity from possible damage caused by the water frost. From these inspections, maps of dimensions, attenuation and velocity were obtained. From the dimension maps, it was noted that the concrete specimens without air entraining agents are more degraded than the specimens with air entraining agents and the edges of the specimens are not defined. These results agree with the surface mass loss results. The decrease of velocity and attenuation after the cycles were more accentuated in concrete without air entrainment and coincides with the results obtained with the monitoring. The attenuation and velocity maps show the microcracking and internal defects in the specimens.

The non-destructive methodology proposed in this work, using ultrasonic velocity measurements and a thermoporometrical model can be an alternative method to evaluate the damage in cementitious materials subjected to F-T cycles. The monitoring of ultrasonic velocity along of the F-T cycles allows one to identify the beginning of damage.

## Figures and Tables

**Table 1.** Properties of constituent's phases.

**Phases**	***C*_11_ (GPa)**	***C*_44_ (GPa)**	***ρ* (kg/m^3^)**
Matrix–Mix A	32.88	8.04	2,175
Matrix–Mix B	36.57	9.55	2,068
Sand/Gravel	86	28	2,600
Water	2.2	0	1,000
Ice	13.33	3.7	920

**Table 2.** Mix proportions and characteristics of the concrete specimens.

**Materials**	**Mix A**	**Mix B**
Cement (kg/m^3^)	450	360
Water (l/m^3^)	180	162
Sand (kg/m^3^)	610	680
Gravel (kg/m^3^)	1,190	1,160
Superplasticizer (by weight of cement)	0.40%	0.30%
Air content (%)	7.5	2.6

**Table 3.** Microstructural characterisation of the concrete specimens.

		**Mix A**	**Mix B**
**Porosity (%)**	Before the cycles	13.1	15.0
	After the cycles	15.9	17.9

**Degree of hydration (%)**	Before the cycles	50.32	55.90
	After the cycles	66.79	53.72

**Density (kg/m^3^)**	Before the cycles	2,504	2,433
	After the cycles	2,437	2,401
